# Multidimensional regulation of transcription factors: decoding the comprehensive signals of plant secondary metabolism

**DOI:** 10.3389/fpls.2025.1522278

**Published:** 2025-03-26

**Authors:** Hongwei Li, Nana Chen, Hongbin Zhang, Delin Xu

**Affiliations:** ^1^ Department of Medical Instrumental Analysis, Zunyi Medical University, Zunyi, Guizhou, China; ^2^ Department of Soil and Crop Sciences, Texas A&M University, College Station, TX, United States; ^3^ Department of Cell Biology, Zunyi Medical University, Zunyi, Guizhou, China

**Keywords:** secondary metabolites, transcription factors, signaling pathways, biosynthesis, mechanisms

## Abstract

Plants synthesize an extensive array of secondary metabolites in response to diverse biotic and abiotic stresses. These metabolites function not only as defensive compounds but also constitute significant sources of nutrition and pharmaceuticals. However, the mechanisms governing the synthesis of these secondary metabolites have long been a central focus of research and continue to pose significant challenges. Transcription factors (TFs), serving as key regulators of secondary metabolite synthesis in plants, exhibit mechanisms of action that are still not fully understood. This review summarizes the latest research advancements on how plant transcription factors mediate the regulation of secondary metabolite biosynthesis through various signaling pathways, including light signaling, hormone signaling, MAPK signaling, the ubiquitin-proteasome pathway, epigenetic regulation, microbial interactions, and climate change. A deeper understanding of the mechanisms regulating transcription factors is expected to provide new insights into the biosynthesis of plant secondary metabolites.

## 1 Introduction

As living chemical factories, plants synthesize a wide variety of secondary metabolites that do not directly participate in primary processes of growth and reproduction but often have significant ecological functions (Zhao et al., 2023b). Over 200,000 primary and secondary metabolites have been identified in plants, most of which are classified as secondary (or specialized) metabolites (Zhan et al., 2022). These secondary metabolites mainly include five major categories: alkaloids, flavonoids, sulfur-containing compounds, terpenes, and fatty acid derivatives, which not only help plants resist pathogens and adapt to environmental stress but also possess antibacterial, anti-inflammatory, anticancer, anti-aging, and antioxidant properties, thus finding wide applications in medicine, food, and cosmetics. Additionally, some secondary metabolites can act as allelochemicals, affecting the growth of neighboring plants and reducing competitive pressure (Hassani et al., 2023).

The therapeutic potential of plant secondary metabolites has long been a research hotspot, many high-value pharmacological components have been isolated from various plants. For instance, artemisinin is used in the treatment of both malaria and cancer (Posadino et al., 2023), while paclitaxel is a prominent anti-cancer drug (Jiang et al., 2024). Secondary metabolites isolated from Polygonati Rhizome (PR) have shown anti-inflammatory, antioxidant, and anti-aging effects (Pan et al., 2024). The antioxidant, anti-inflammatory, and antibacterial properties of terpenes and phenolics are used to promote wound healing (Romo-Rico et al., 2022). In recent years, plant secondary metabolites have become important raw materials in the pharmaceutical industry, aiding in the development of new drugs for human disease treatment. However, the synthesis mechanisms of these secondary metabolites have long been a research hotspot and challenge. Given the significance of secondary metabolites in plant physiology and industrial applications, understanding their biosynthetic regulatory mechanisms has become exceedingly crucial. Among these mechanisms, transcription factors serve as the central regulators of gene expression.

**Figure 1:**
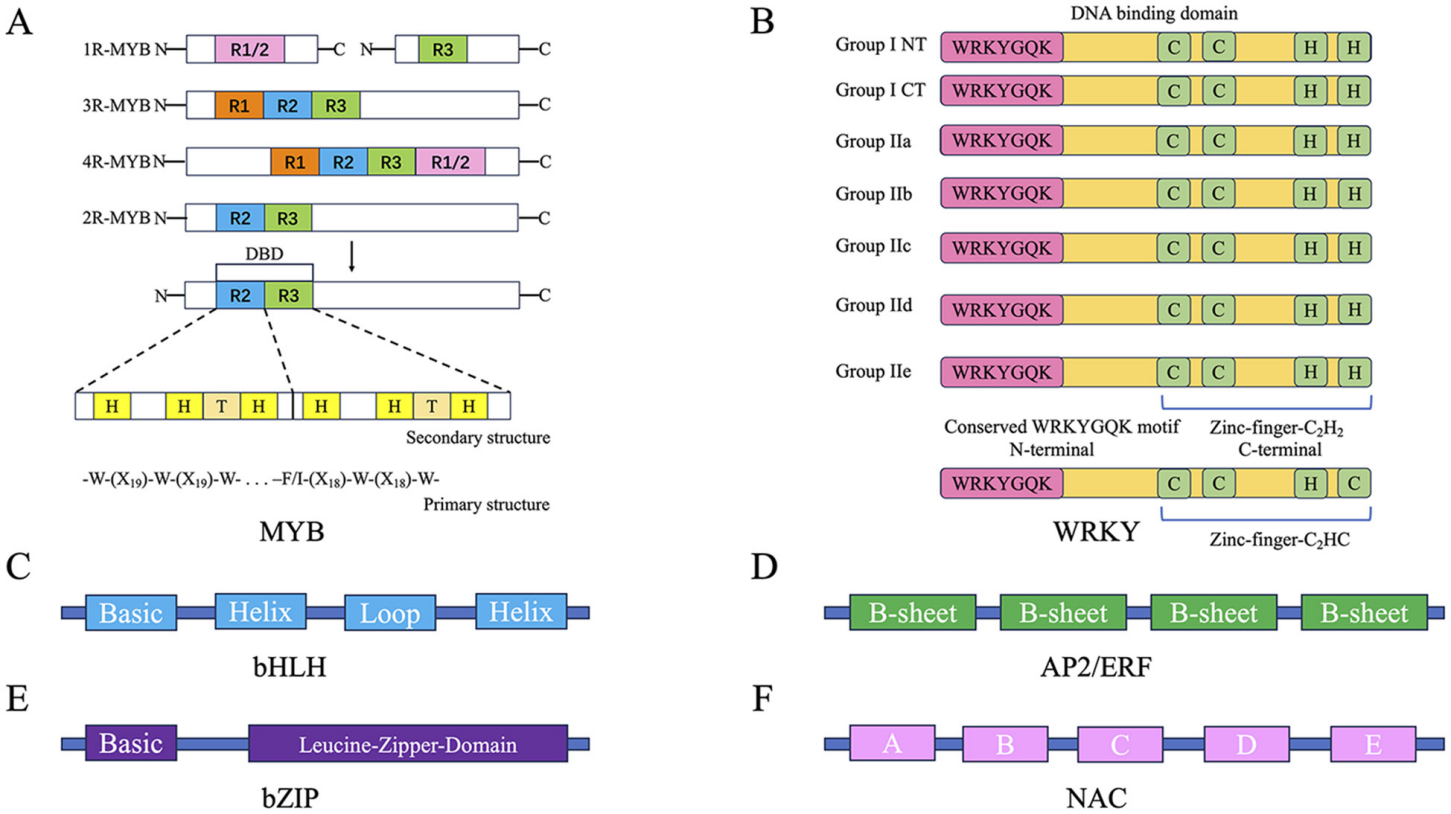
Structural characteristics of typical transcription factors. **(A)** Structural features of four subclasses of MYB transcription factors, including primary and secondary structures. **(B)** Zinc finger structural characteristics and conserved domain sequences of the seven subclasses of WRKY transcription factors. **(C)** Helical structure of bHLH transcription factors. **(D)** Basic structure of AP2/ERF transcription factors. **(E)** Basic structure of bZIP transcription factors. **(F)** Basic structure of NAC transcription factors, with the white letters **(A–E)** representing different subdomains.

Transcription factors (TFs) are currently the most extensively studied regulators of plant secondary metabolism, controlling the expression of genes encoding specific enzymes at the transcriptional level (Deng et al., 2024). Transcription factor families such as MYB, WRKY, bHLH, and bZIP have been shown to be widely involved in the biosynthesis of various plant secondary metabolites. They perform different biological functions due to their distinct DNA-binding domains, transcriptional activation domains, and transcriptional repression domains (**Figure 1**). For example, transcription factors such as CsMYB8 and CsMYB99, EuWRKY30, SmbHLH60 and SmMYC2, and PpbZIP44 are involved in the synthesis of anthocyanins, eucommia rubber, phenolic acids, and flavonoids in *Camellia sinensis*, *Eucommia ulmoides*, *Salvia miltiorrhiza*, and *Pyrus pyrifolia*, respectively (Li et al., 2022; Zhang et al., 2024b; Liu et al., 2022a; Wang et al., 2023a). Other transcription factors such as NAC (Zhang et al., 2021a), TCP (Liu et al., 2023b), EIN3 (Song S. et al., 2022), HY5 (An et al., 2024), ZCT (Zhang et al., 2024c), MADS-box (Tantisuwanichkul et al., 2024), YABBY (Kayani et al., 2021; Wang et al., 2016), HD-Zip (Gao et al., 2021), SPL (Wang et al., 2020), BBX (He et al., 2023c), and AP2/ERF (Zhang et al., 2021b) have also been reported to be involved in the biosynthesis of plant secondary metabolites.

However, the molecular mechanisms by which transcription factors participate in the accumulation of secondary metabolites are not yet fully understood. In recent years, various environmental factors (light, low temperature), endogenous signals (hormones), and various regulatory factors (ncRNAs) have been shown to be important in regulating the activity of transcription factors related to secondary metabolism. For example, light signals regulate the expression of the transcription factor MYB24, activating genes related to flavonoid synthesis and thereby promoting the biosynthesis of flavonoids in grapes (Zhang et al., 2023a). Plant hormones are important signaling molecules within plants, such as jasmonic acid (JA) compounds, which are considered derivatives of fatty acids. Under the mediation of the transcription factor AchMYC2, the transcription of *AchPAL*, *AchC4H*, and *Ach4CL* in *Actinidia chinensis* is activated, promoting JA-mediated suberin polyphenolic accumulation (Wei et al., 2022). Additionally, other signaling pathways involving transcription factors, such as MAPK signaling, microbial regulation, epigenetic regulation, and climate change, can also affect the accumulation of secondary metabolites.

This study conducted a systematic search across several prominent databases, including PubMed, Scopus, Web of Science, and Google Scholar, using keywords such as “transcription factors”, “plant secondary metabolites”, “signaling pathways”, “biosynthetic mechanisms”, “bioactive compounds”, and “pharmacological components”. We selected high-quality and high-impact references from the past three to five years to gather a comprehensive array of relevant research. This review highlights the critical role of transcription factors in mediating various signaling pathways that regulate the synthesis of secondary metabolites. In addition, this paper analyzes the current issues and challenges in the research on the regulation of plant secondary metabolism synthesis, yield and quality, and the treatment of human diseases, and proposes forward-looking solutions, including the application of artificial intelligence and deep learning in the regulation of secondary metabolite synthesis in plants, the use of heterologous synthesis techniques to enhance the yield of secondary metabolites, and the challenges associated with the application of secondary metabolites in clinical medicine. These studies are expected to provide new strategies for the future regulation of plant secondary metabolite synthesis and the development of new plant-based drugs.

## 2 Biosynthetic pathways of plant secondary metabolites

Most secondary metabolites have relatively small molecular weights, typically less than 1500 Da, and are often synthesized in specific anatomical structures, cell types, or organelles, specifically for production and storage (Li et al., 2024b). Plant secondary metabolites include terpenoids, phenylpropanoids, nitrogen-containing compounds, sulfur-containing compounds, and so on. They are primarily synthesized using precursors produced by primary metabolic pathways, but not directly participating in plant growth, development, and reproduction. Instead, they are produced under specific conditions and serve functions such as defending against pathogen infection, responding to environmental stress, and protecting against ultraviolet radiation damage. And it has been confirmed that these metabolites contain a large number of bioactive components which could be widely used in the pharmaceutical, food, cosmetics, and biofuel industries.

### 2.1 Terpenoids

Terpenoids are the largest class of natural products produced by terrestrial plants. Although a few terpenoids, such as gibberellins, are plant hormones that play key roles in regulating growth and development, the vast majority of terpenoids are specialized or secondary metabolites involved in various plant-environment interactions (Jia et al., 2022). They are widely distributed in animals, plants, fungi, and bacteria (Luo et al., 2024). Terpenoids can be classified into monoterpenes, sesquiterpenes, diterpenes, triterpenes, tetraterpenes, and polyterpenes based on the number of isoprene units (Wang et al., 2023b).

Monoterpenes are the largest class of plant secondary metabolites, consisting of terpene compounds formed by two isoprene units, with each unit containing five carbon atoms. They are primarily found in various plants, including those in the *Lamiaceae* family (Wojtunik-Kulesza, 2022). Common monoterpenes include limonene (Araujo-Filho et al., 2021) and eucalyptol (Ogueta et al., 2022). Research on monoterpenes has become relatively extensive, with applications spanning multiple fields, including the prevention of atherosclerosis (Yang et al., 2023), pest control (Liu et al., 2022b), and cancer treatment (Zielinska-Blajet et al., 2021). Additionally, monoterpenes have been reported to possess the ability to regulate energy metabolism, exhibit anti-diabetic and anti-obesity effects, and modulate gut microbiota (de Alvarenga et al., 2023).

Sesquiterpenes are compounds composed of three isoprene structural units and are widely distributed among various angiosperms, some gymnosperms, and mosses. Due to their rich content of various anticancer active components, sesquiterpenes have garnered significant attention. For example, artemisinin, a natural sesquiterpene lactone extracted from the traditional Chinese medicinal herb sweet wormwood (*Artemisia annua* L.), is considered a cornerstone in the current treatment of malaria (Wen et al., 2024; White and Chotivanich, 2024). Similarly, diterpenes, triterpenes, and tetraterpenes are formed by the combination of multiple isoprene (C5) units in various ways, and they generally exhibit anticancer activity and potential for treating other diseases.

**Figure 2:**
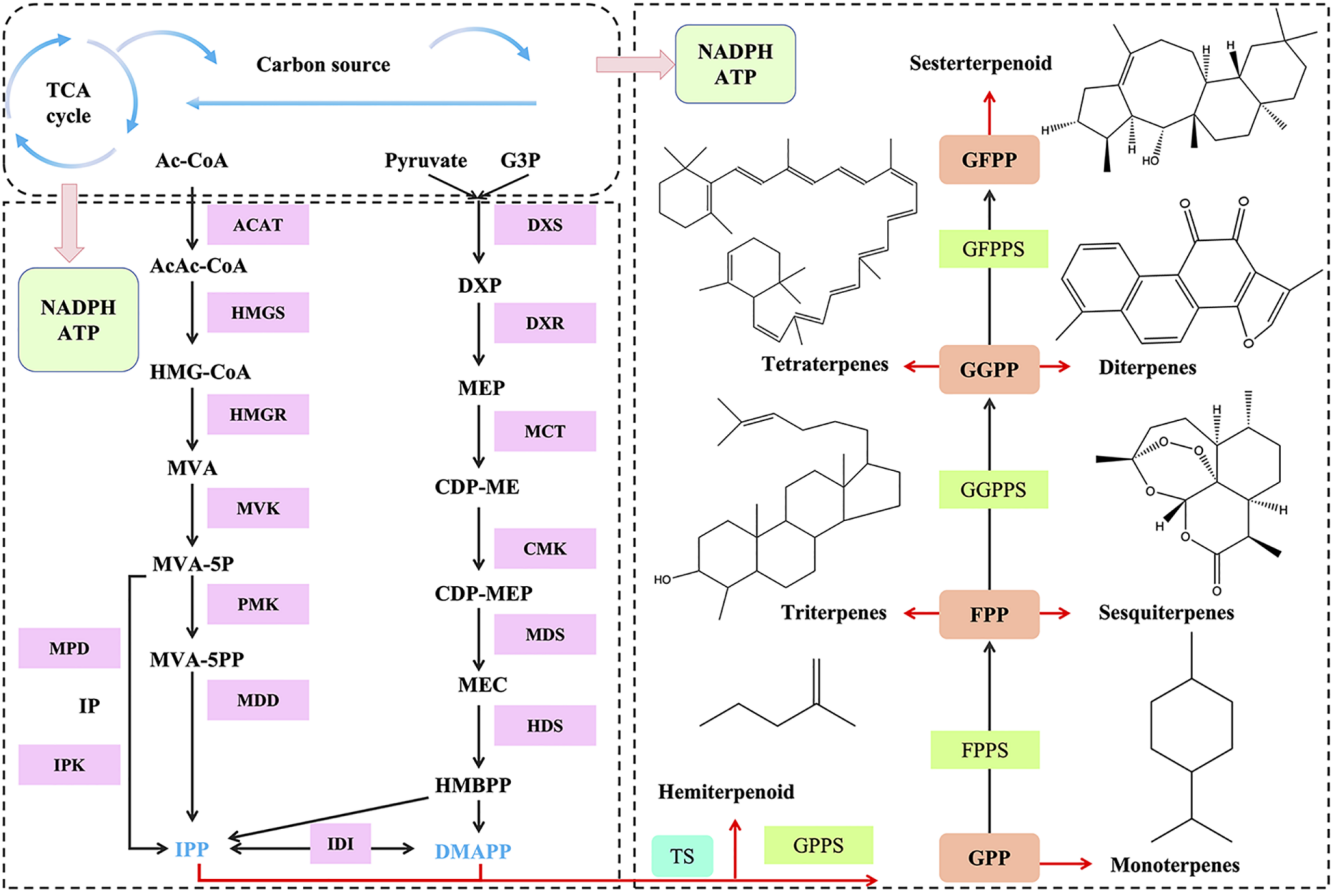
Terpene synthesis pathway. ACAT, acetyl-coenzyme A C-acetyl­ transferase; HMGS, 3-hydroxy-3-methylglutaryl coenzyme A synthase; HMGR, 3-hydroxy-3-methyl glutaryl coenzyme A reductase; PMK, phosphomevalonate kinase; MPD, mevalonate pyrophosphate decarboxylase; MVA, mevalonic acid; DXS, 1-deoxy-D-xylulose 5-phosphate synthase; DXR, 1-deoxy-D-xylulose-5-phosphate reductoisomerase; MEP, 2-C-methyl-D-erythritol 4-phosphate; IPP, isopenteny pyrophosphate; DMAPP, dimethylallyl diphosphate; GPPS, geranyl diphosphate synthase; GPP, geranyl diphosphate; GGPPS, geranylgeranyl pyrophosphate syn­thase; GGPP, geranylgeranyl diphosphate; CDP-ME,4-(cytidine5’-diphospho)-2-C-methyl-D-erythritol; CDP-MEP, 4-diphosphocytidyl-2-C-methyl-d-erythritol-2-phosphate; G3P,glyceraldehyde 3-phosphate; HMG-CoA,3-hydroxy-3-methylglutaryl-CoA; IDI, isopentenyl diphosphate isomerase; GFPP, geranylfarnesyl diphosphate; GFPPS, geranylfarnesyl diphosphate synthase; FPP, farnesyl pyrophosphate; TS, terpene synthase; MDD, mevalonate diphosphate decarboxylase; MVA-5-P, mevalonate 5-phosphate; MVA-5-PP, mevalonate 5-diphosphate; MPD, mevalonate phosphate decarboxylase; IPK, isopentenyl phosphate kinases; IP, isopentenyl phosphate; MDS, 2-C-methyl-D-erythritol 2,4-cyclodiphosphate synthase; HDS, (E)-4-hydroxy-3-methylbut-2-enyl diphosphate synthase; MCT, 2-C-methyl-D-erythritol 4phosphate synthase; CMK, CDP-ME-kinase; HMBPP, 4-hydroxy-3-methylbut-2-enyl-diphosphate.

Terpenes are primarily synthesized through two pathways: the mevalonate (MVA) pathway and the methylerythritol-4-phosphate (MEP) pathway (Lv et al., 2024) (**Figure 2**). The MVA pathway originates in eukaryotes and occurs in the cytoplasm, endoplasmic reticulum, and peroxisomes, while the MEP pathway originates in prokaryotes and operates in plastids (Hilal et al., 2024). IPP (isopentenyl pyrophosphate) and its interconvertible isomer DMAPP (dimethylallyl diphosphate) are two important precursor substances produced by both the MVA and MEP pathways. In the MVA pathway, terpenoids are synthesized from acetyl-CoA and converted into mevalonate pyrophosphate (MVA-PP) through a series of condensation and reduction reactions. MVA-PP eventually undergoes decarboxylation to form IPP, which can be isomerized to DMAPP by isopentenyl pyrophosphate isomerase (IDI). Terpenoids are also synthesized via the MEP pathway, which starts with the condensation of glycolysis intermediates glyceraldehyde 3-phosphate (GAP) and pyruvate. Through further phosphorylation, cyclization, and reductive isomerization, IPP and DMAPP are formed. IPP and DMAPP are further converted into monoterpene precursors (10-C geranyl pyrophosphate, GPP), sesquiterpene precursors (15-C farnesyl pyrophosphate, FPP), and diterpene precursors (20-C geranylgeranyl pyrophosphate, GGPP) under the catalysis of various prenyltransferases (Dinday and Ghosh, 2023). Finally, these precursors are converted into monoterpenes, sesquiterpenes, triterpenes, diterpenes, and tetraterpenes under the catalysis of various enzymes.

Cannabinoids, tanshinones, artemisinin, paclitaxel, and ginsenosides are common terpenoid compounds that possess pharmacological properties promoting human health and have shown great potential in treating human diseases. For example, artemisinin has antimalarial effects; ginkgolides have neuroprotective, reparative, and anti-ischemic stress effects; cannabinoids have anxiolytic and anesthetic effects, etc (Wang et al., 2023b). Tanshinone I has various therapeutic effects, including cardiovascular protection, anticancer, anti-inflammatory, and anti-neurodegenerative properties (Ke et al., 2023). Terpenoids are also commonly used as lead compounds for anticancer and antitumor drugs. For instance, paclitaxel plays a significant role in antitumor therapy by inducing oxidative stress and is considered one of the most successful natural anticancer drugs.

### 2.2 Nitrogen compounds

Nitrogen-containing compounds are another class of plant secondary metabolites, including alkaloids, non-protein amino acids, and polyamines. Previous studies have reported that nitrogen-containing secondary metabolites obtained from endophytes of medicinal plants exhibit potentially beneficial anticancer, antimicrobial, antifungal, antiviral, and antimalarial activities for human health (Khan et al., 2024). Alkaloids are low molecular weight nitrogen-containing compounds primarily derived from amino acids. Alkaloids are mainly classified into terpenoid indole alkaloids, isoquinoline alkaloids, and tropane alkaloids.

Alkaloids are also widely used for enhancing neural excitation and for their antitumor properties. For instance, colchicine, vinblastine, and vincristine are well-known for their antitumor effects by inhibiting tubulin polymerization. Alkaloids also exhibit neuroprotective properties, showing great potential in the treatment of neurodegenerative diseases.

### 2.3 Phenylpropanoids

Phenylpropanoid metabolism is one of the most important metabolic pathways in plants, producing over 8,000 metabolites that are involved in plant growth and development, as well as plant-environment interactions. The phenylpropanoid compounds produced mainly include lignin, flavonoids, lignans, phenylpropanoid esters, hydroxycinnamic acid amides, and sporopollenin (Dong and Lin, 2021).

**Figure 3:**
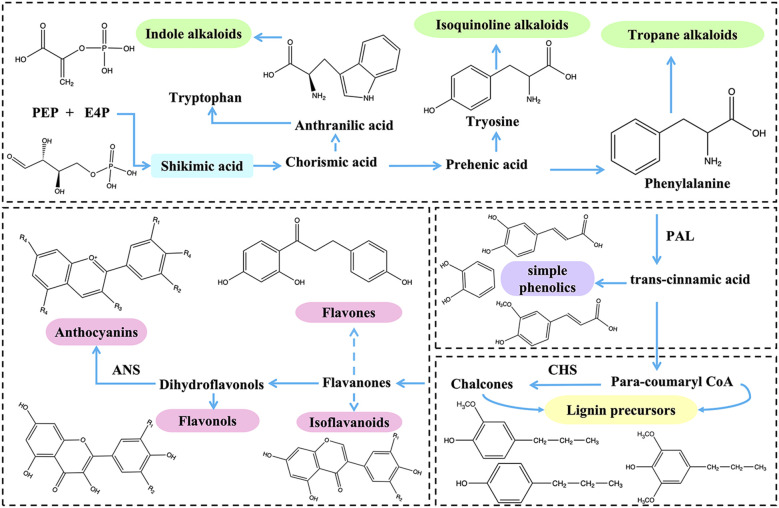
Synthetic pathways of flavonoids and alkaloids. R1, R2, R3 and R4 are substituted by different groups to form different compounds. ANS, anthocyanidin synthase; CHS, chalcone synthase; PAL, phenylalanine ammonia-lyase.

The phenylpropanoid metabolic pathway, as a downstream reaction of the shikimic acid pathway, primarily involves the biosynthesis of phenylalanine. Phenylalanine can synthesize various polyphenolic secondary metabolites through multiple enzymatic reactions, including flavonoids, lignin, anthocyanins, and isoflavones. In the phenylpropanoid metabolic pathway, the key rate-limiting enzymes for the formation of these secondary metabolites are phenylalanine ammonia-lyase (*PAL*), chalcone synthase (*CHS*), and anthocyanin synthase (*ANS*) (Li et al., 2023) (**Figure 3**). Phenylalanine ammonia-lyase (*PAL*) is a key enzyme that catalyzes the conversion of L-phenylalanine to trans-cinnamic acid, participating in the phenylpropanoid biosynthetic pathway in plants and microorganisms. It plays a crucial role in connecting primary and secondary metabolism (Fan et al., 2022). Chalcone synthase (*CHS*), classified as a type III polyketide synthase, is also a critical enzyme in the phenylpropanoid pathway, primarily responsible for generating precursors for flavonoid biosynthesis (Mahajan et al., 2023). Anthocyanin synthase (*ANS*) is a downstream enzyme in the anthocyanin biosynthetic pathway and serves as a rate-limiting enzyme, catalyzing the conversion of colorless anthocyanins to colored anthocyanins. This process is essential for the coloration of plants and plays a significant role in their stress resistance (Feng et al., 2023).

Flavonoid metabolism is an important branch of phenylpropanoid metabolism and produces the largest class of polyphenolic metabolites, containing approximately over 6,000 compounds (Dong and Lin, 2021). According to their structure, flavonoids can be classified into seven subgroups: flavonols, flavones, isoflavones, anthocyanins, flavanones, flavanols, and chalcones. Flavonoids are widely present in fruits, vegetables, and other cereal crops. They have beneficial biochemical effects on various diseases (such as cardiovascular diseases, atherosclerosis) and other biological activities (such as anti-inflammatory, anti-aging) (Shen et al., 2022). In plants, flavonoid compounds are usually found in flowers, leaves, and seeds. They provide coloration to flowers to attract animals and protect plants from biotic stressors (such as herbivores, bacteria, fungi) and abiotic environmental stressors (such as UV absorption). Additionally, flavonoids play multiple roles in plant-microbe interactions (Wang et al., 2022a).

Anthocyanins are one of the most extensively studied flavonoids, primarily found in plants, fruits, and vegetables. They typically impart red, purple, and blue colors to flowers and fruits. Their potential anticancer and antiviral activities have been well-documented (Khan et al., 2024). Anthocyanins can function as both exogenous and endogenous antioxidants, with different types showing varying efficiencies in scavenging reactive oxygen and nitrogen species. Besides their antioxidant properties, anthocyanins are important for vision and the prevention of cardiovascular diseases. Moreover, anthocyanins have potential in preventing and treating intestinal diseases such as inflammatory bowel disease (IBD), irritable bowel syndrome (IBS), and colorectal cancer (Liu et al., 2024a).

### 2.4 Sulfur-containing compounds

Sulfur-containing compounds (SCC) are important secondary metabolites widely distributed in plants, especially in the *Brassicaceae* family. Isothiocyanates and glucosinolates are common sulfur-containing compounds. These compounds not only play a crucial role in the interactions between plants and pests but also exhibit various therapeutic effects in the human body, including chemoprotective anti-cancer effects, regulation of the endocrine system, and improvement of sexual function (He et al., 2023b).

**Figure 4:**
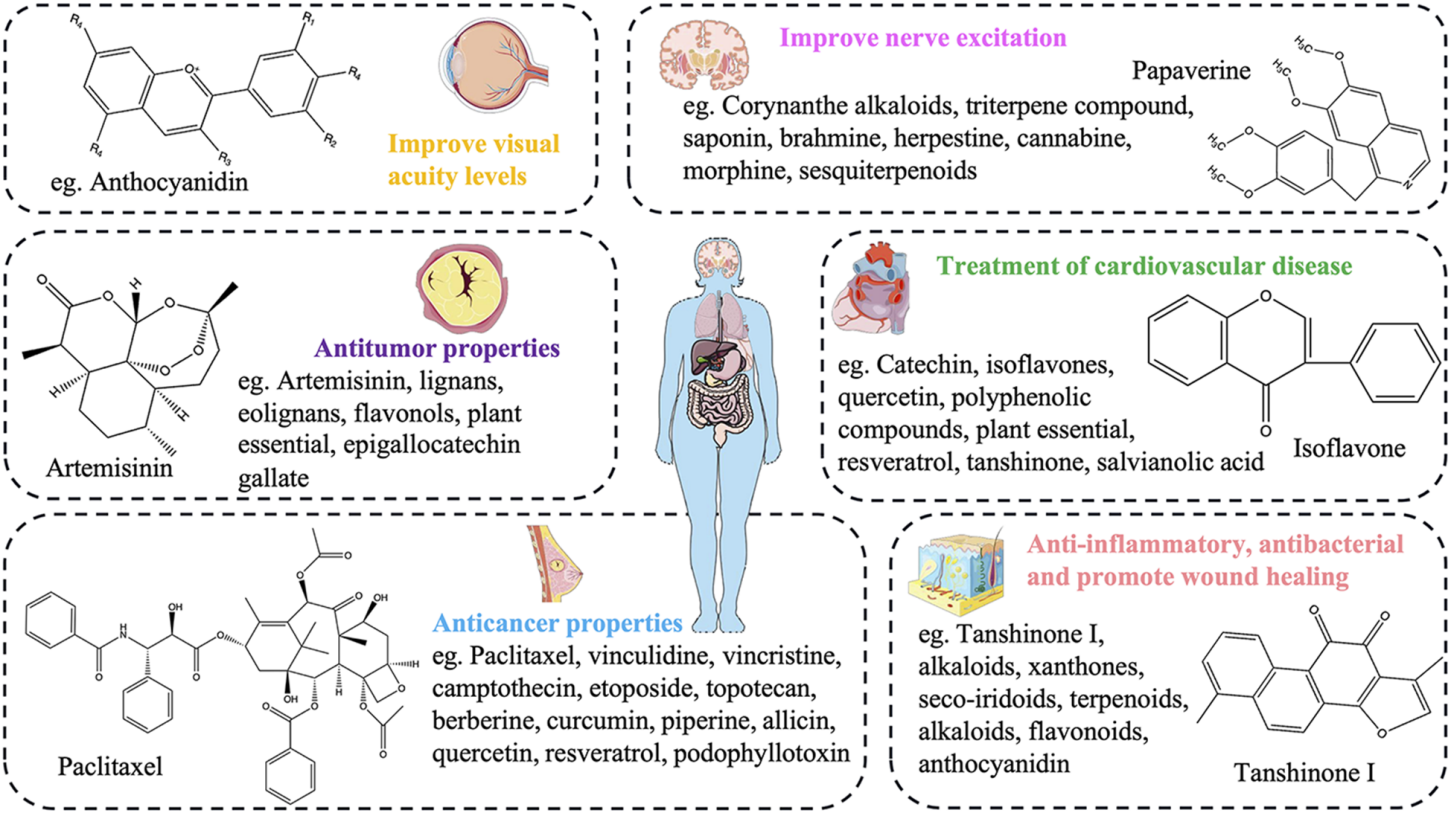
The potential pharmacological effects of plant secondary metabolites in the treatment of human diseases.

Overall, the diversity of plant secondary metabolites is incredibly vast, resembling a massive and mysterious treasure trove. These rich and diverse secondary metabolites encompass countless unique components and substances. In the treatment of various human diseases, they have shown immense potential (**Figure 4**), whether in breakthroughs in drug development or in providing innovative therapeutic approaches. They hold invaluable significance and promise, opening up broad prospects and a hopeful future for the treatment of human diseases.

## 3 Transcription factors mediate multiple signaling pathways to influence the accumulation of secondary metabolites

Transcription factors play a crucial role in regulating the synthesis of plant secondary metabolites. They influence the accumulation of secondary metabolites by forming specific signal transduction pathways through exogenous signals (light signaling), endogenous signals (hormones), and various regulatory factors.

### 3.1 Light signaling

Light is one of the crucial environmental factors affecting plant growth and development. The impact of light on plants is mainly reflected in three aspects: light quality, light intensity, and photoperiod. Changes in photoperiod affect plant flowering and secondary metabolism.

HY5 is a major transcription factor in the light signal transduction pathway, and AaHY5 has been shown to regulate the expression of downstream transcription factor genes such as *AaMYB108* to promote artemisinin biosynthesis (Liu et al., 2023a). AaBBX21 is a transcription factor identified in *Artemisia annua* that can form a complex with AaHY5, enhancing transcriptional activation capability of AaHY5. Meanwhile, light signals enhance the accumulation and activation of AaHY5. Together, they synergistically activate the promoters of downstream genes *AaGSW1*, *AaMYB108*, and *AaORA*, thereby enhancing artemisinin accumulation (He et al., 2024a).

In eggplant (*Solanum melongena* L.), the transcription factor SmMYB5 is a key factor responding to JA signals and positively regulating anthocyanin synthesis. It interacts with the bHLH transcription factor SmTT8 to form a complex that activates key enzyme genes in the anthocyanin synthesis pathway. However, the protein stability of SmMYB5 is regulated by light signals. Light signals promote its degradation through the interaction between SmCOP1 and SmMYB5, thereby reducing anthocyanin accumulation (Li et al., 2024a).

CsbHLH89 activates the transcription of key structural genes in the anthocyanin synthesis pathway, such as *CsCHS*, *CsFLS*, and *CsDFR*, by directly interacting with the G-box elements in their promoter regions. Under the guidance of light signals, CsHY5 can bind to the promoter region of *CsbHLH89*, further enhancing anthocyanin biosynthesis in *Camellia sinensis* (Zhang et al., 2024a).

It is evident that light signals are crucial in regulating the synthesis of plant secondary metabolites and are closely associated with the involvement of various transcription factors.

### 3.2 Hormone signaling

Changes in plant secondary metabolism may be caused by variations in plant hormones. Abscisic acid (ABA), ethylene (ETH), strigolactone (SL), jasmonic acid (JA), salicylic acid (SA), Indole-3-acetic acid (IAA), gibberellins (GAs), and are considered key regulators of the biosynthesis, transport, accumulation, and release of plant secondary metabolites (Lv et al., 2024). Transcription factors respond to hormone signals, altering their activity to regulate the expression of genes related to secondary metabolism, thereby affecting the accumulation of secondary metabolites.

#### 3.2.1 Abscisic acid

Abscisic acid (ABA) is a sesquiterpene hormone named for its role in accelerating leaf abscission. Reports also indicate that transcription factors can respond to ABA and influence the accumulation of secondary metabolites. For instance, ABI5 is a member of the bZIP family and plays a crucial role in ABA signal-mediated apple anthocyanin biosynthesis. The overexpression of MdABI5 significantly increases the anthocyanin content of plants, and moreover, this process depends on MdbHLH3. The interaction between MdABI5 and MdbHLH3 enhances the transcriptional regulation of target genes and the binding ability of the MYB1-bHLH3 complex (An et al., 2021). Similarly, the overexpression of ABA-responsive element-binding factor 3 (ABF3) increases the accumulation of flavonoids and phenolic acids in transgenic soybeans under drought stress (Kumari et al., 2024). Research in *Salvia miltiorrhiza* has also demonstrated that abscisic acid (ABA) enhances the kinase activity of SmAPK1, promoting the phosphorylation and degradation of the negative regulatory factor SmbZIP4. This process alleviates the inhibition of key genes involved in the biosynthesis of tanshinone, thereby increasing its content (Zhu et al., 2024).

#### 3.2.2 Ethylene

Ethylene (ETH) is present in all parts of the plant, including stems, leaves, flowers, roots, seeds, and fruits. It is a plant growth regulator widely used to accelerate the ripening of flowers and fruits (Baharudin and Osman, 2023). In recent years, the regulatory role of ethylene in plant secondary metabolism has been gradually revealed. Ethylene regulates the production of chalcone isomerase, chalcone synthase, and flavonol synthase, controlling flavonol biosynthesis with the involvement of the myeloblastosis (MYB12) transcription factor (Baharudin and Osman, 2023). During the ripening process of apples, ethylene plays an important role. On the one hand, it activates the positive regulatory factors MdMYB1 and MdEIL1, and on the other hand, it induces the expression of the negative regulatory factor MdMYB17, thereby forming a complex regulatory network. In this network, MdMYB17 can directly suppress the expressions of MdMYB1 and MdEIL1, while MdMYB1 and MdEIL1 can also activate the expression of MdMYB17, thus forming a negative feedback cycle (Wang et al., 2022c). This complex regulatory network jointly conducts fine regulation on the synthesis of anthocyanin and the formation of fruit color.

Notably, a recent study has found that there can be crosstalk between ethylene and melatonin (MT), which subsequently affects the synthesis of isoflavones in soybean sprouts. The interaction between melatonin (MT) and ethylene in soybean sprouts stimulates isoflavone biosynthesis through a series of molecular processes. Ethylene, introduced via exogenous ethephon treatment, enhances isoflavone content but also induces oxidative stress, reducing sprout growth. Melatonin mitigates this stress by activating antioxidant defense systems, including increasing the activities of key enzymes like superoxide dismutase, catalase, and peroxidase. This action helps maintain cellular integrity and reduce harmful reactive oxygen species (ROS). Additionally, MT promotes isoflavone biosynthesis by upregulating the expression of genes involved in the phenylpropanoid pathway, particularly those encoding enzymes like phenylalanine ammonia-lyase (*PAL*), cinnamic acid 4-hydroxylase (*C4H*), and 4-coumarate coenzyme A ligase (*4CL*). The synergistic effect of MT and ethylene ultimately leads to higher isoflavone content in the sprouts, promoting both antioxidant capacity and secondary metabolite accumulation (Tian et al., 2024). These findings provide a solid theoretical basis for further understanding the interactions between plant hormones and the synthesis of flavonoids.

#### 3.2.3 Strigolactone

Strigolactones (SLs), a novel class of carotenoid-derived plant hormones discovered in 2008, possess diverse functions. SLs serve not only as regulators of plant architecture but also as signaling molecules in rhizosphere communication, playing a crucial role in the plant’s response to abiotic stresses (Visentin et al., 2024). In recent years, SLs have garnered significant attention for their pivotal roles in regulating plant development and plant-environment interactions. SLs exhibit a remarkable dual function: they act as both exogenous signaling molecules and endogenous hormones, thereby enhancing the plant’s ability to adapt to various growth conditions and stresses (Guercio et al., 2023).

A recent study suggests that strigolactones engage in crosstalk with other hormones to induce anthocyanin synthesis. Strigolactones initiate the signaling pathway by binding to the D14 receptor, which facilitates MdPRT1-mediated degradation of MdSMXL8, thereby releasing MdAGL9 to activate MdHY5 expression, ultimately enhancing anthocyanin biosynthesis. Conversely, gibberellins (GA) disrupt the interaction between MdSMXL8 and MdAGL9 through their repressor MdRGL2a. This research underscores the critical role of the intricate regulatory network among plant hormones in anthocyanin synthesis (An et al., 2024).

#### 3.2.4 Jasmonates

Jasmonates are a class of oxylipins, a family of compounds that includes jasmonic acid (JA) and its derivatives, such as methyl jasmonate (MeJA) and amino acid conjugates. As endogenous hormonal signals, jasmonates play a key role in plant defense by inducing the production of defense compounds and regulating stress responses.

The bHLH transcription factor MYC2, a pivotal regulator within jasmonate signaling and plant specialized metabolism, exhibits sensitivity to repression by JASMONATE-ZIM-domain (JAZ) proteins and is co-activated by the mediator subunit MED25. The activity of the transcription factor NtMYC2a is inhibited by JAZ proteins and co-activated by MED25, and mutations at specific sites on NtMYC2a can alter its interaction with these signaling components, thereby regulating the expression of synthase genes such as *QPT*, *PMT*, and *BBL*, which are involved in the synthesis and accumulation of alkaloids like nicotine in plants (Hou et al., 2023). Additionally, another study showed that the transcription factor NtMYB305a can bind to the JA-responsive element GAG region of the *NtPMT1a* promoter, promoting the transcription and translation of this gene, thereby enhancing nicotine biosynthesis in *Nicotiana tabacum* (Bian et al., 2022). Methyl jasmonate can also induce a series of physiological and biochemical changes in plants, including promoting the synthesis and accumulation of certain secondary metabolites. The transcription factor PcMYB62 is upregulated under MeJA induction, and its overexpression in *Polygonum cuspidatum* significantly reduces the content of resveratrol in the leaves (Lin et al., 2024). These studies indicate that transcription factors play an important role in responding to hormone signals to regulate the accumulation of secondary metabolites.

An interesting report shows that jasmonates, as elicitors of plant secondary metabolism, can reprogram metabolic pathways, promoting the expression of genes in secondary metabolite biosynthesis pathways and inducing stress-related metabolites in defense genes. Additionally, jasmonates do not act alone; they interact with other plant hormones such as auxin, gibberellins (GA), salicylic acid (SA), brassinosteroids (BR), ethylene (ETH), and abscisic acid (ABA). This interaction is mediated by transcription factors such as bHLH, MYB, ERF, WRKY, and regulatory co-factors like JAZ, DELLA, and AUX/IAA (Khan et al., 2024). Research has shown that the crosstalk between JA and other plant hormones in regulating SM (secondary metabolites) production is mediated by the MYC2 TF and JAZ repressors (Delgado et al., 2021). For instance, by means of yeast two-hybrid, luciferase complementation imaging and *in vitro* experiments, found that there is an interaction between the *CwJAZ4/9* gene and the CwMYC2 transcription factor in *Curcuma wenyujin*, and through transgenic hairy roots and transcriptome analysis, it clarified that these genes play a negative regulatory role in jasmonic acid-mediated terpene biosynthesis (Wang et al., 2024).

#### 3.2.5 Other plant hormones

Salicylic acid (SA), indole-3-acetic acid (IAA), and gibberellic acid (GA) also contribute to the accumulation of secondary metabolites, but the transcription factors involved are not fully identified. SA is an important plant growth regulator widely used to promote the synthesis of high-value nutrients in plants. Exogenous SA treatment significantly improved the growth and yield of *Lilium* and increased the content of soluble sugars, soluble proteins, ascorbic acid (AsA), and colchicine in the leaves. Additionally, under SA treatment, the expression patterns of transcription factors and hormone-related DEGs (differentially expressed genes) associated with plant hormone signal transduction changed (Miao et al., 2024). Exogenous IAA treatment stimulated the transcription levels of several key enzyme genes in the tanshinone biosynthesis pathway, thereby increasing the content of tanshinones (Zhang et al., 2023b). Transcription factor families such as AP2/ERF and WRKY are sensitive to auxin treatment, and they may be involved in regulating lateral root development and secondary metabolite biosynthesis by directly or indirectly regulating the expression of target genes. Exogenous gibberellin treatment can increase root length and weight by upregulating the biosynthesis genes of auxin (IAA) and gibberellin (GA). Some transcription factors related to plant growth and anthocyanin accumulation, such as MYB, bHLH, WRKY, and CYP, are significantly upregulated in transgenic lines (Aktar et al., 2022). Melatonin triggers the expression of the transcription factor CcPCL1, which directly binds to the promoter of *CcF3’H-5*, upregulating its expression and consequently boosting the biosynthesis of luteolin (Song Z. et al., 2022).

Additionally, studies have shown that brassinosteroids (BR) play a significant role in the synthesis of plant secondary metabolites. MdJa2 belongs to the STMADS11 subfamily of the MADS-box family, and its expression is positively regulated by brassinosteroid signaling. Under the influence of the BR signaling activator BL, the expression level of MdJa2 increases, which subsequently binds directly to the promoter regions of key genes in the anthocyanin and proanthocyanidin biosynthetic pathways (such as *MdANS*, *MdMYB9*, and *MdMYB12*), thereby repressing their transcription. Furthermore, MdJa2 interacts with another key transcription factor in the BR signaling pathway, MdBZR1, to form the MdJa2-MdBZR1 complex, which significantly enhances the ability to bind to downstream gene promoters, thereby further reinforcing the repression of anthocyanin and proanthocyanidin biosynthetic pathways. This regulatory mechanism establishes a delicate balance within the plant, ensuring that anthocyanins and proanthocyanidins accumulate at optimal levels to maintain the color and nutritional value of the fruit (Su et al., 2022).

In conclude, plant hormones act as signaling molecules that activate a series of stress-related signaling pathways, promoting the expression of related genes and thereby regulating the synthesis of secondary metabolites. During this process, the activity of transcription factors related to secondary metabolism is also affected. Interestingly, studies have shown that hormone signaling molecules do not act independently; they can crosstalk or synergize with each other to influence the accumulation of secondary metabolites. For instance, A recent study shows that overexpression of *AabHLH113* can increase the content of artemisinin, and the RNAi of *AabHLH113* can significantly reduce the content of artemisinin. However, AabHLH112 induced by JA and AabZIP1 induced by ABA can directly bind to the promoter of *AabHLH113*, thereby affecting the biosynthesis of artemisinin, which indicates that the expression of *AabHLH113* is induced by JA and ABA (Yuan et al., 2023). Therefore, it is very necessary to deeply explore whether the transcription factors or genes induced by different hormones independently form regulatory modules or form regulatory modules through cooperation. Furthermore, supplementing plant hormones and other signaling molecules related to the production of secondary metabolites can enhance crop resistance, which helps promote sustainable food production and nutritional security. However, a comprehensive understanding of the regulatory interactions between plant hormones and other signaling molecules under environmental stress requires further molecular and genetic research.

### 3.3 MAPK signaling

In plants, the mitogen-activated protein kinase (MAPK) signaling pathway is one of the key mechanisms regulating cellular responses to external environmental stimuli. It not only participates in controlling plant growth and development but also plays a significant role in the regulation of secondary metabolism in plants.

In recent years, research has reported the involvement of MAPK signaling in the regulation of secondary metabolite synthesis in plants, with various transcription factors participating in the regulation. In *Catharanthus roseus*, CrMAPKKK1 can sequentially activate and phosphorylate CrMAPKK1 and CrMAPK3/6. CrMAPK3/6 can enhance the activation of upstream *CrMYC2* and *ORCA* gene clusters, thereby regulating the expression of terpenoid indole alkaloid (TIA) synthesis genes (Liu et al., 2023b). SmMAPK3 interacts with transcription factors including SmMYB36, SmMYB39, SmMYB111, SmPAP1, and SmAREB1, which directly target and regulate key enzyme genes involved in tanshinone synthesis such as *SmCPS1*, *SmCYP76AH1*, and *SmDXR* (Xie et al., 2020). Further research has revealed that the MAPK signaling pathway interacts with plant hormone signaling pathways such as jasmonic acid (JA), ethylene (ETH), and abscisic acid (ABA), jointly regulating plant secondary metabolism. For instance, JA is one of the important defense hormones in plants; it can enhance plant resistance to pests and diseases by activating the MAPK signaling pathway and promoting the synthesis of some resistance-related secondary metabolites. Therefore, a deeper understanding of the specific mechanisms of MAPK signaling in plant secondary metabolism not only helps to elucidate the intrinsic regulatory networks of plant responses to environmental stress but also provides potential avenues for enhancing the synthesis and accumulation of plant secondary metabolites through genetic engineering.

### 3.4 Ubiquitin-proteasome pathway

**Figure 5:**
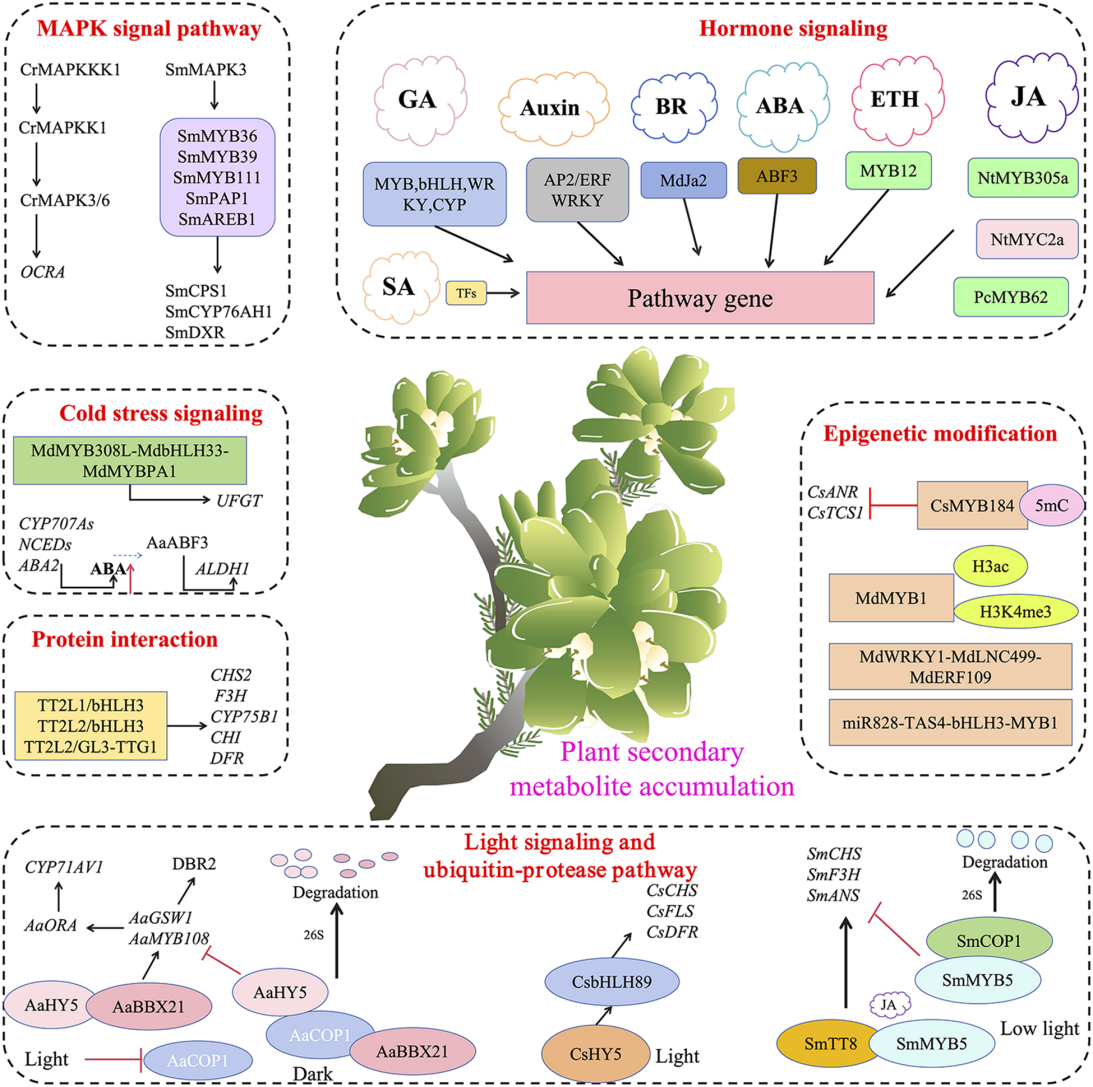
Transcription factors mediate various signal transduction and participate in the accumulation of plant secondary metabolites.

The ubiquitin-proteasome system (UPS) is ubiquitously present in eukaryotic cells and serves as a crucial pathway for regulating the homeostasis of endogenous proteins. In this pathway, protein substrates destined for degradation are recognized by ubiquitin ligases, which mediate their ubiquitination, ultimately promoting their degradation by the proteasome (Wang et al., 2022b). Ubiquitination is a major post-translational modification of proteins, and the ubiquitin-26S proteasome system (UPS) comprises ubiquitin-activating enzyme E1, ubiquitin-conjugating enzyme E2, and ubiquitin ligase E3. Among these, E3 ubiquitin ligases are the key determinants of substrate specificity (Zhao et al., 2023a). Recent studies have found that the ubiquitin-proteasome pathway plays an important regulatory role in the accumulation of secondary metabolites (**Figure 5**).

Previous studies have shown that under the guidance of light signals, the AaBBX21 and AaHY5 complex can promote the expression of genes in the artemisinin biosynthetic pathway, thereby indirectly enhancing the accumulation of artemisinin. However, under dark conditions, the function of the AaBBX21 and AaHY5 complex is diminished. AaCOP1, a core repressor in the light signaling pathway, is an E3 ubiquitin ligase that interacts with the Val-Pro (VP) motif through its WD-40 domain, ubiquitinating interacting proteins and degrading them via the 26S proteasome pathway. AaCOP1 can interact with AaBBX21 and AaHY5, leading to their degradation via the 26S proteasome pathway, thereby reducing artemisinin accumulation. Light can inhibit the expression of AaCOP1, enhancing the binding ability of AaHY5 to downstream gene promoters (He et al., 2024a). VvPUB26 is an E3 ubiquitin ligase in grape (*Vitis vinifera*). VvWRKY24 acts as a negative regulator by binding to the promoters of *DFR* and *LAR*, inhibiting their transcription. The interaction between VvPUB26 and the transcription factor VvWRKY24 regulates proanthocyanidin biosynthesis by promoting the ubiquitination of VvWRKY24, which is then recognized and degraded by the 26S proteasome (Zhao et al., 2024). Therefore, the mechanism of secondary metabolite accumulation mediated by the ubiquitin-proteasome pathway is of great significance for a deeper understanding of the molecular mechanisms regulating plant secondary metabolism.

### 3.5 Epigenetic modifications

DNA methylation, histone modifications, and non-coding RNA regulation are crucial components in the regulation of secondary metabolite synthesis in plants.

DNA methylation and histone modifications have been shown to regulate gene expression by altering chromatin structure, thereby affecting anthocyanin biosynthesis (Khan and Abbas, 2023). A recent study revealed that the activity of the CsMYB184 transcription factor in tea plants is regulated by 5mC methylation. Changes in the methylation status of its encoding gene affect its expression levels, thereby influencing the expression of flavonoid and caffeine secondary metabolism pathways *CsANR* and *CsTCS1* in tea plants (Han et al., 2024). These findings highlight the importance of dynamic DNA methylation in seasonal-dependent secondary metabolism and provide new insights for improving tea quality. Combined application of targeted metabolite analysis, whole-genome bisulfite sequencing, and transcriptome sequencing revealed that elevated DNA methylation levels are associated with increased expression of various epigenetic regulatory genes, including *SmCMT2*, *SmDDM1*, *SmAGO4*, and *SmDRM1*, affecting the accumulation of tanshinones and salvianolic acids. Furthermore, the expression levels of many genes involved in the biosynthesis of tanshinones and salvianolic acids, such as *SmCPS5*, *SmCYP71D464*, *SmGGPPS1*, *SmGPPS*, *SmHPPR*, and *SmHPPD*, are altered due to hypermethylation (He et al., 2024b). This indicates that DNA methylation plays a crucial role in regulating the accumulation of tanshinones and phenolic acids. Post-translational modifications of histones influence the binding of transcription factors to chromatin by altering the affinity between histones and DNA strands. Among these, H3K9ac, H3ac, and H3K4me3 play critical roles in the biosynthesis of plant anthocyanins. For example, light-induced apple pigmentation is associated with increased H3ac and H3K4me3 activity at the *MdMYB1* promoter (Liu et al., 2023b).

Non-coding RNAs (ncRNAs), including microRNAs (miRNAs) and long ncRNAs (lncRNAs), regulate plant secondary metabolism not only at the transcriptional level but also provide nuanced regulation at the post-transcriptional level (Deng et al., 2024). For example, miRNAs have been demonstrated to form targeted regulatory modules with transcription factor genes, inhibiting their expression and thereby indirectly regulating the activity of transcription factors. This, in turn, can modulate the expression of downstream genes, leading to the enhancement or suppression of phenolic compound accumulation. In *Malus domestica*, the miR828-TAS4-bHLH3-MYB1 regulatory module forms a feedback loop involved in anthocyanin metabolism (Zhang et al., 2020). LncRNAs directly or indirectly participate in the regulation of transcription factor-mediated secondary metabolite synthesis. In the fruit of sea buckthorn (*Hippophae rhamnoides*), virus-induced gene silencing (VIGS) of LNC1 and LNC2 revealed that these lncRNAs can act as endogenous target mimics (eTMs) of miR156a and miR828a, inhibiting *SPL9* and activating *MYB114* expression, thereby increasing and decreasing anthocyanin content, respectively. Besides acting as eTMs, lncRNAs can also directly mediate the expression of TFs or enzymes dedicated to anthocyanin biosynthesis. For example, MdLNC499 is regulated by MdWRKY1, which in turn induces MdERF109, forming a MdWRKY1-MdLNC499-MdERF109 cascade that regulates the expression of downstream anthocyanin-related genes during the early stages of apple coloration (Ma et al., 2021).

Epigenetic regulation has shown its important role in regulating the synthesis and accumulation of secondary metabolites. However, despite the remarkable progress made in research, there are still some areas that need to be further explored. For example, existing research mostly focuses on single epigenetic modifications, such as DNA methylation, histone modification and non-coding RNA regulation, lacking a systematic study on the synergistic regulatory mechanism of multiple epigenetic modifications. In the future, a multi-level and multi-dimensional regulatory network should be constructed, and its complex mechanism should be comprehensively analyzed by using high-throughput sequencing and systems biology methods, while exploring its potential in plant breeding and biotechnological applications.

### 3.6 Microbial regulation

In recent years, an increasing number of studies have shown a close relationship between plant-associated microorganisms (including rhizosphere and endophytic microbes) and the synthesis of plant secondary metabolites. Microorganisms significantly influence the accumulation of secondary metabolites in plants by regulating hormone levels, nutrient uptake, precursor supply, as well as the expression of genes and transcription factors (Lv et al., 2024). For example, microorganisms can produce specific chemical signals to influence the synthesis and signaling of jasmonic acid (JA), salicylic acid (SA), gibberellins (GA), and ethylene (ETH). Moreover, transcription factors play a crucial role in the signaling mechanisms of these hormones. For example, MYC2 serves as a bHLH transcription factor and is a crucial regulatory component within the jasmonic acid (JA) signaling pathway. When JA signals are present, JAZ proteins undergo degradation, liberating the MYC2 transcription factor, which subsequently binds to the promoter regions of downstream genes to regulate gene expression, thus mediating plant growth, development, and defense responses (Mu et al., 2025). Furthermore, MYC2 participates in both synergistic and antagonistic interactions between JA and various hormones, including gibberellins (GA), abscisic acid (ABA), indole-3-acetic acid (IAA), and ethylene (ETH). MYC2 is also capable of interacting with melatonin (MT), brassinosteroids (BR), and resveratrol to regulate a multitude of aspects related to plant growth and development.

Recent studies on *Ginkgo biloba* have demonstrated that the MYC2 transcription factor plays a pivotal role in regulating the synthesis of the secondary metabolite terpene trilactone (TTL) via the jasmonic acid (JA) signaling pathway. Under standard or low levels of JA, JAZ proteins associate with MYC2, thereby inhibiting its activity. When methyl jasmonate (MeJA) is externally applied, JA levels rise, resulting in the degradation of JAZ proteins and the liberation of MYC2.The liberated MYC2 can specifically recognize and bind to the G-box elements (e.g., CACGTG) within the promoter region of the key gene *GbGGPPS*, which is involved in TTL synthesis, thereby activating its expression. The enzyme encoded by *GbGGPPS* represents a critical branching point in the TTL synthesis pathway, and its upregulated expression directly facilitates the synthesis and accumulation of TTL (Zheng et al., 2024). Likewise, transcription factors, including MYCs, MYBs, bHLH, and AP2/ERF, can respond to synergistic signals stemming from endogenous fungal stimuli involving gibberellins (GA), JA, ethylene (ETH), and salicylic acid (SA), thereby directly or indirectly regulating the expression of genes or transcription factors linked to synthesis, consequently impacting the production of paclitaxel (Cao et al., 2022). Furthermore, the external inoculation of microorganisms can stimulate the synthesis of various hormones within plants, which subsequently mediates the synthesis of secondary metabolites through the regulation of transcription factors. For instance, Inoculation with *P. fluorescens* can stimulate the production of GA and CK and trigger responses in JA and ETH, thereby upregulating the expression of CrWRKT2. This promotes the expression of genes in the TIA biosynthesis pathway, including geraniol-10-hydroxylase (*G10H*), deacetylvindoline-4-O-acetyltransferase (*DAT*), tabersonine 16-hydroxylase (*T16H*), and *C. roseus* peroxidase (*CrPRX*) (Ahmadzadeh et al., 2022). Thus, the significant role of microorganisms in the synthesis of plant secondary metabolites is of great importance for the construction of plant-microbe reactors aimed at enhancing metabolite yields.

### 3.7 Climate change

**Figure 6:**
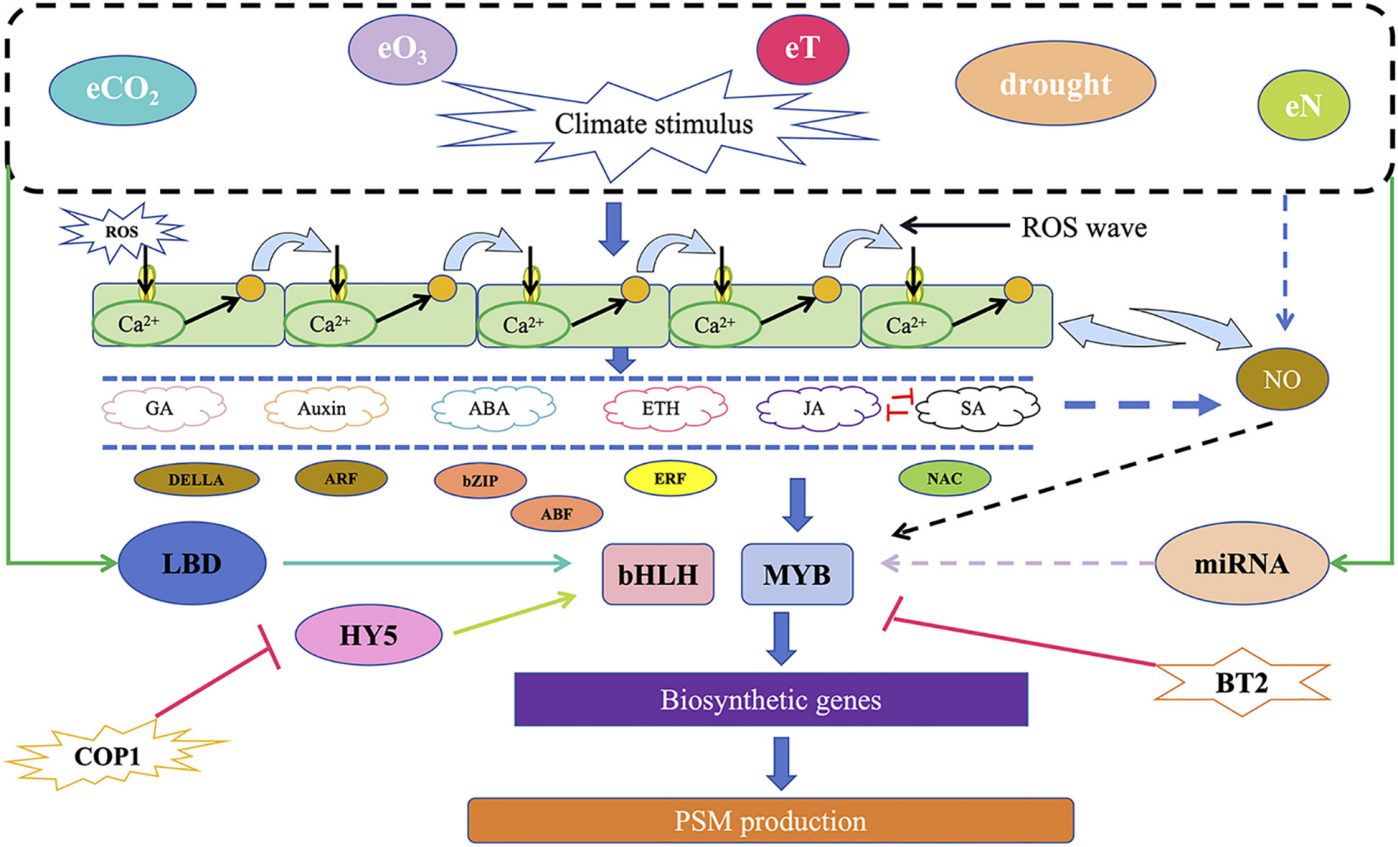
A schematic model of the potential molecular mechanisms by which various environmental and climatic stimuli regulate the synthesis of plant secondary metabolites (Modified from Sun and Fernie, 2024).

Environmental climate change is a significant factor affecting the synthesis of plant secondary metabolites. Elevated carbon dioxide concentration (eCO_2_), elevated ozone concentration (eO_3_), nitrogen deposition (eN), increased temperature (eT), drought, and various combinations of these climate factors can influence the accumulation of secondary metabolites. Sun et al. reviewed the mechanisms by which transcription factors such as MYB and bHLH respond to these factors and influence the accumulation of plant secondary metabolites, providing valuable insights for understanding the regulation of secondary metabolism under environmental climate change (Sun and Fernie, 2024) (**Figure 6**).

In addition, cold stress negatively impacts plant growth and agricultural productivity, typically classified into two levels: low temperature (0–15°C) and freezing (<0°C). Low temperature stress reduces the fluidity of plant cell membranes, disrupts protein stability, inhibits enzyme activity, and affects gene expression and protein synthesis (Ding et al., 2024). Studies have shown that low temperatures can also affect plant secondary metabolism, with the greatest impact on the synthesis of flavonoids and terpenoids (He et al., 2023a). Under cold stress, plants activate relevant transcription factors (TFs), promoting the accumulation of secondary metabolites. The bHLH and MYB transcription factor families play crucial roles in responding to cold signal transduction. The MYB and bHLH families in the MBW regulatory complex system are activated under cold stress conditions. bHLH33 is a bHLH transcription factor that positively regulates cold tolerance and anthocyanin biosynthesis. Under cold stress conditions, MdMYB308L, bHLH33, and MdMYBPA1 form a targeted regulatory module that promotes the expression of the *UFGT* gene involved in anthocyanin synthesis (He et al., 2023a).

Additionally, cold stress stimulates the expression of plant hormone biosynthesis genes, increasing endogenous hormone levels (ethylene, jasmonic acid, abscisic acid), inducing the expression of hormone response factors, and indirectly promoting the biosynthesis of secondary metabolites. Cold stress upregulates the expression levels of genes in the ABA biosynthesis pathway, including *CYP707As*, *NCEDs*, and *ABA2*. The ABA-responsive transcription factor AaABF3 can directly bind to the promoter of the key artemisinin biosynthesis gene *ALDH1*, activating its expression and thereby regulating artemisinin accumulation (He et al., 2023a).

Interestingly, there exists a certain degree of crosstalk between light signals and cold signals. For instance, CRY2, as a blue light receptor, can perceive blue light and undergo conformational changes, thereby activating its biological functions. The blue light-activated CRY2 stabilizes the HY5 protein, which is a key transcription factor that can directly bind to the promoter regions of anthocyanin biosynthesis genes, activating the expression of flavonoid biosynthetic enzyme genes and subsequently promoting the synthesis of anthocyanins in *Arabidopsis thaliana* (Ding et al., 2024). Obviously, stress conditions are the key factors to stimulate plants to produce secondary metabolites. Therefore, it is very necessary to study the mechanism of environmental conditions on the accumulation of plant secondary metabolites.

In short, the transcription factors appear to act as “central hubs” in regulating the biosynthesis of secondary metabolites by modulating the expression of related genes. They not only actively regulate the expression of metabolism-related genes but also passively gain transcriptional activity in response to stimuli from other regulatory factors. Interestingly, there are certain correlations among the factors influencing the activity of transcription factors, seemingly forming a network-like complex regulatory system that affects the production of secondary metabolites.

## 4 Prospect

Current research on the regulation of plant secondary metabolism is primarily centered on the analysis of secondary metabolic pathways to elucidate the biosynthetic processes of specific compounds. This involves identifying key enzymes, transcription factors, and other regulatory proteins. Furthermore, the roles of non-coding RNAs, epigenetic modifications, environmental stressors, microbial interactions, and endogenous hormones in regulating plant secondary metabolism are actively being explored. However, plant secondary metabolic pathways are intricately interwoven with primary metabolism, leading to complex interactions that make their regulatory mechanisms and biosynthetic pathways challenging to fully elucidate. Moreover, validating the functions of metabolism-related genes and proteins in plants presents considerable challenges. Additionally, enhancing the yield and stability of secondary metabolites in plant cultivation remains an ongoing challenge.

### 4.1 Applications of deep learning and artificial intelligence in plant secondary metabolism

In recent years, multi-omics technologies (transcriptomics, proteomics, metabolomics), gene editing technologies, bioinformatics technologies, single-cell analysis technologies, and synthetic biology technologies have become the main tools for secondary metabolism research. Based on these technologies, network models of most plant secondary metabolisms have been constructed, and metabolic pathways have been reconstructed through synthetic biology methods to explore the synthesis of new products. The application of gene editing technologies such as CRISPR/Cas9 has identified the biological functions of various metabolism-related synthetic genes. Although the application of these technologies has solved some major challenges in the study of plant secondary metabolism synthesis regulation, fully elucidating the synthesis mechanisms of secondary metabolites remains a complex and novel topic. Therefore, these technologies should be further utilized to deeply explore the mysteries of plant secondary metabolism. It is necessary to continuously optimize multi-omics technologies, expand the application scope of gene editing technologies, increase bioinformatics research and development, widely apply single-cell analysis technologies, and overcome the limitations of synthetic biology technologies. Through coordinated development and comprehensive application, we can accelerate the resolution of the challenges in the regulation of plant secondary metabolism synthesis, bringing more innovative results and development opportunities to agriculture, medicine, and chemical industries.

Furthermore, the application of emerging technologies is an inevitable trend for the future. With the advent of large language models like ChatGTP o1-mili, DeepSeek and Claude3.5, artificial intelligence and deep learning technologies are also being applied to botanical research. Recent studies have shown breakthrough results of the AlphaFold 3 model in predicting protein structures, especially in predicting protein-ligand interactions, with accuracy surpassing previous methods (Abramson et al., 2024). This paves the way for the *de novo* design of proteins with specific functions. The application of deep learning in plant secondary metabolism shows a promising prospect. Recently, a bio-retrosynthesis model integrating deep learning and retrieval technology, READRetro, was reported, specifically for predicting the biosynthetic pathways of natural products. READRetro demonstrates significant capability in predicting the biosynthetic pathways of both known and unknown secondary metabolites. For known metabolites such as monoterpene indole alkaloids, READRetro accurately recapitulates established biosynthetic routes by integrating a generative model with a reaction retriever, which retrieves conserved metabolic reactions from a large database. In the case of unknown pathways, such as that of menisdaurilide, READRetro proposes plausible biosynthetic routes by leveraging its generative module to predict novel reactions, thereby expanding the scope of biosynthetic pathway elucidation. This dual approach of generation and retrieval enables READRetro to effectively navigate the complex chemical space of plant natural products, providing valuable insights into their biosynthetic processes (Kim et al., 2024). Therefore, in the future, deep learning algorithms and big data analysis can be utilized to more efficiently analyze plant secondary metabolic pathways and the regulatory mechanisms of related genes. These technologies can process and analyze vast amounts of genomic, transcriptomic, and metabolomic data, revealing complex biological networks and metabolic pathways.

It is noteworthy that a recent study indicates that the artificial transcription factor SmMYB36-VP16 can significantly increase the content of tanshinones and phenolic acids in *Salvia miltiorrhiza* (Jia et al., 2024). Therefore, deep learning algorithms and artificial intelligence can be utilized to design specific transcription factors that target genes related to secondary metabolic pathways, thereby enhancing the production of secondary metabolites. Additionally, artificial intelligence can be used to predict changes in plant secondary metabolites under different environmental conditions, optimize plant breeding and gene editing strategies, thereby enhancing the medicinal and economic value of plants. This will greatly advance the research on the regulation of plant secondary metabolism, bringing new breakthroughs to the fields of agriculture, medicine, and biotechnology.

### 4.2 Improve secondary metabolite synthesis through heterologous synthesis of plants and microorganisms

As the largest natural product factories in nature, plants can synthesize a rich diversity of secondary metabolites. These metabolites have important applications in the fields of medicine, food, and agriculture. However, the natural yield of these metabolites is often low who can no longer meet the high demand of humans and the market, limiting their commercial development and application. Therefore, developing new strategies to enhance the synthesis of plant secondary metabolites is imperative.

It has shown that specific rhizosphere and endophytic microorganisms can significantly increase the synthesis of plant secondary metabolites (Lv et al., 2024). This discovery lays a solid foundation for heterologous synthesis of plants and microorganisms. Utilizing heterologous synthesis of plants and microorganisms to enhance the synthesis of secondary metabolites is a promising research direction.

At present, research on the heterologous synthesis of plant secondary metabolites based on various microorganisms has achieved considerable success. For example, in the field of exploring the heterologous synthesis of secondary metabolites by microorganisms, the research team of Li successfully achieved the efficient synthesis of Zealexin A1 in Saccharomyces cerevisiae by using fine engineering strategies. They increased the yield of Zealexin A1 by 261.7 times by screening and modifying key enzymes, as well as optimizing fermentation conditions, providing strong empirical support for increasing the yield of secondary metabolites (Li et al., 2024c). Similarly, Winegar and colleagues achieved efficient production of verazine in Saccharomyces cerevisiae through a series of engineering modifications. These modifications included a reconstructed biosynthetic pathway and regulation of cholesterol biosynthesis, ultimately achieving a verazine yield of 83 ± 3 μg/L in a 5-liter bioreactor (Winegar et al., 2024). It is evident that the utilization of microbial heterologous synthesis can effectively yield specific plant secondary metabolites, with production levels that may even exceed those achieved through conventional extraction methods. In comparison to traditional extraction methods, microbial heterologous synthesis presents numerous advantages, including reduced production cycles, conservation of land resources, and customizable culture conditions.

In addition, microbial heterologous synthesis has other potential advantages. It can modify microorganisms through genetic engineering and metabolic engineering to further improve the yield and quality of secondary metabolites. At the same time, microbial heterologous synthesis can also achieve structural modification and functional optimization of secondary metabolites, providing the possibility for developing compounds with higher biological activity and medicinal value.

### 4.3 Challenges in understanding and applying plant secondary metabolites in medicine

In recent years, the antioxidant, anti-inflammatory, antitumor, immune-regulating, antibacterial, and antiviral functions of plant secondary metabolites have been widely described. However, their specific mechanisms of action have not been systematically elucidated, leading to doubts and misunderstandings about their therapeutic potential.

While some studies have deeply illustrated the mechanisms by which secondary metabolites treat human diseases, indeed bringing relief to patients. For example, past research has shown that paclitaxel has a unique antitumor mechanism. It can stabilize microtubules, inhibit mitosis, and induce apoptosis in cancer cells, thereby preventing their proliferation and exerting anticancer effects (Jiang et al., 2024). A recent study reported that artemisinin targets mitochondrial protease LONP1, promotes the binding of LONP1 to its substrate *CYP11A1*, accelerates the degradation of *CYP11A1*, thereby inhibiting ovarian androgen synthesis, reducing androgen levels in patients with polycystic ovary syndrome (PCOS), and improving menstrual cycles and ovarian polycystic changes (Liu et al., 2024b). These research outcomes demonstrate the great potential of plant secondary metabolites in treating human diseases.

Although numerous plant secondary metabolites have been shown to possess therapeutic effects on human diseases, their specific mechanisms of action remain incompletely understood, thereby limiting their further application and development. Moreover, ensuring the quality control of these medicinal compounds presents significant challenges. Consequently, future efforts should focus on establishing stringent raw material procurement standards and employing advanced analytical techniques such as high-performance liquid chromatography (HPLC) for the quantitative detection of key active components, which are essential measures to ensure product consistency. Additionally, the stability of secondary metabolites presents a significant challenge in quality control. By optimizing storage conditions and packaging materials, as well as conducting systematic stability testing, the shelf life of products can be effectively extended. Furthermore, the lack of unified quality standards complicates the comparison of products from different manufacturers. Participating in the development of industry standards and referencing guidelines from international pharmacopoeias can contribute to the establishment of a unified quality control system.

In summary, a comprehensive understanding of the biosynthetic mechanisms of plant secondary metabolites is crucial for the development of new drugs and innovative strategies for treating human diseases. Although the specific mechanisms by which plant secondary metabolites exert therapeutic effects are not yet fully elucidated, we anticipate the identification of additional therapeutic targets in the future. Continued dedication to the research of traditional Chinese medicine will undoubtedly yield significant contributions to medical advancements and human well-being.
